# Microenvironment changes in arteriovenous malformations after stereotactic radiation

**DOI:** 10.3389/fnhum.2022.982190

**Published:** 2022-12-15

**Authors:** Timothy H. Ung, Katherine Belanger, Ayesha Hashmi, Vashisht Sekar, Antonio Meola, Steven D. Chang

**Affiliations:** ^1^Department of Neurosurgery, Stanford University, Palo Alto, CA, United States; ^2^Department of Neurosurgery, University of Colorado School of Medicine, Aurora, CO, United States

**Keywords:** arteriovenous malformation, microenvironment changes in AVM, radiation for AVM, stereotactic radiosurgery for AVM, changes after radiation in AVMs

## Abstract

Cerebral arteriovenous malformations are dysplastic vascular tangles with aberrant vascular dynamics and can result significant morbidity and mortality. A myriad of challenges are encountered when treating these lesions and are largely based on nidal size, location, and prior hemorrhage. Currently, stereotactic radiosurgery is an accepted form of treatment for small to medium sized lesions and is especially useful in the treatment of lesions in non-surgically assessable eloquent areas of the brain. Despite overall high rates of nidal obliteration, there is relatively limited understand on the mechanisms that drive the inflammatory and obliterative pathways observed after treatment with stereotactic radiosurgery. This review provides an overview of arteriovenous malformations with respect to stereotactic radiosurgery and the current understanding of the mechanisms that lead to nidal obliteration.

## Introduction

Arteriovenous malformations of the brain are dysplastic tangles of low-resistance channels between arteries and veins. These are vascular lesions characterized by a web of abnormal vessels that directly shunt high flow blood from the feeding arteries to draining veins. They undergo dynamic changes in growth, vascular remodeling, and regression, which makes these vascular lesions difficult to characterize and can result in intracranial hemorrhage. Ruptured of AVMs can carry significant morbidity and patients are at risk for future hemorrhages. Unruptured AVMs are controversial as the morbidity and mortality of treatment may exceed that of the AVM's natural history. Increased use of non-invasive cranial imaging has also increased the prevalence of incidentally discovered lesions and studies have been aimed at investigating the natural history of AVMs in a more comprehensive nature. Patient presentation differs from patient to patient and is dependent on the size, location, and venous drainage.

Management of ruptured and unruptured AVMs necessitates a multidisciplinary team and treatment strategies include observation, microsurgical resection, endovascular embolization, and stereotactic radiosurgery. The goal of treatment is complete obliteration of the AVM with preservation of neurologic function. SRS has become increasingly important in the management of AVMs and can offer favorable outcomes in AVMs located in eloquent and deep brain areas. Understanding the mechanisms that drive nidal obliteration and microvascular changes is critical and further understanding will continue to advance treatment strategies. In this review, we aim to provide a brief overview of the AVMs with a focus on SRS and a comprehensive review of the current understood mechanisms that drive the microvascular changes observed after radiation treatment. To fully understand the mechanisms that drive microenvironment and biological changes after radiosurgery, we will first review the current evidence for treatment of AVMs with radiosurgery followed by a review of AVM biology and AVM microenvironment and biological changes after radiosurgery.

## Epidemiology

AVMs occur at an incidence of 0.69–1.42 per 100 000 as described by a collection of population-based studies from the literature (Steiner et al., 1972; Jessurun et al., 1993; Brown et al., 1996; Hofmeister et al., 2000; Choi and Mohr, 2005; Laakso and Hernesniemi, 2012; Nagy et al., 2012; Mohr et al., 2014; Cohen-Inbar et al., 2016; Osbun et al., 2017). Historically, incidence was primarily based on patients with symptomatic presentation, but increased use of non-invasive cranial imaging has led to a paralleled increase in the overall prevalence of AVMs in modern population-based studies. The risk of hemorrhage for untreated unruptured AVMs is 1–5% per year as reported by natural-history studies and an increased risk of rupture is observed in patients with a history of prior AVM rupture. This risk of re-hemorrhage in ruptured AVM patients is greatest within the first year of the patient's initial AVM hemorrhage (Jessurun et al., 1993; Brown et al., 1996; Zhu et al., 1997). In all, 5–25% of all AVM hemorrhages are fatal and the susceptibility for rupture is related to the vascular architecture, intrinsic flow dynamics, venous drainage characteristics, nidus location, and relative size (Mast et al., 1997; Hernesniemi et al., 2008; da Costa et al., 2009; Kim et al., 2014). In addition to hemorrhage, seizure is a common presenting symptom and is more common in patients with cortically based lesions, especially within the temporal lobe. Up to one third of patients with AVMs can present with seizures and post hemorrhagic development of seizures can occur in up to one-half of all patients (Josephson et al., 2011, 2012; Garcin et al., 2012). Other focal neurologic symptoms can be present in patients and is largely dependent on lesion location, size, and vascular flow.

## Radiosurgery for AVMs

Initial radiosurgery for AVMs was marked by successful obliteration of the lesion and demonstrated overall safety (Steiner et al., 1972; Colombo et al., 1987; Betti et al., 1989). SRS technologies have advanced remarkably, and minimally invasive SRS has become a standard management option for AVMs. It is particularly useful for lesions located in deep or eloquent regions with high surgical risks. AVM obliteration with LINAC-based radiosurgery is safe and effective and achieved complete AVM obliteration in about 60–80% of cases with an approximate obliteration time of 3–5 years depending on various factors (Paul et al., 2014; Pollock et al., 2016; Ding et al., 2017a; Starke et al., 2017). The most prominent predictors of AVM success included AVM size, volume, radiation dose, number of draining veins, and patient age (Ding et al., 2016). Stereotactic radiosurgery has been found to be particularly effective for small to medium-sized AVMs with diameter of <30 mm and is especially effective for small lesions in eloquent areas of the brain (Ding et al., 2017b; Chan et al., 2019; Karlsson et al., 2019; Peciu-Florianu et al., 2020). For larger AVMs with a volume >10 cm^3^, a staged fractionated approach may be used (Franzin et al., 2016). Additional treatment options for larger and more complex lesions employ a combination of stereotactic radiosurgery with endovascular embolization and open cranial resection.

Given the diversity of AVMs, scoring systems have been developed aimed at predicting outcomes after SRS. Two important scoring systems include the modified Radiosurgery-Based AVM score (RBAS) and the Virgina Radiosurgery AVM Scale (VRAS). The RBAS includes nidus volume, location, and patients age and is used to calculate AVM obliteration without a new neurologic deficit (Pollock and Flickinger, 2002; Raffa et al., 2009). Alternatively, the VRAS score is composed of the nidal volume, location, and includes prior hemorrhage and outcomes are defined as lesional obliteration without post radiation hemorrhage or permanent radiation-induced complications (RIC) (Starke et al., 2013) (Table 1).

**Table 1:** Both the Modified radiosurgery-based AVM score and Virgina radiosurgery AVM scale are accepted scoring systems used with radiosurgical treatment of AVMs.

**SRS AVM Scores**		
**Modified radiosurgery-based AVM score**	[0.1 × nidus volume (cm ^3^)] + [0.02 × patient age (years)] + [0.5 × nidus location score]	AVM obliteration without new neurologic deficit
	Deep locations = 1	Score total
	• Basal ganglia	≤1.00 = 62%
	• Brainstem	>1.00 – 2.00 = 53%
	• Thalamus	<2.00 = 32 %
	Other locations = 0	
	• Frontal	
	• Temporal	
	• Parietal	
	• Occipital	
	• Intraventricular	
	• Corpus callosum	
	• Cerebellar	
**Virginia radiosurgery AVM scale**	AVM volume	Favorable outcome with AVM obliteration with no
	<2 cm^3^ = 0 points	post-radiation hemorrhage or symptomatic RIC
	2–4 cm^3^ = 1 point	Score total
	>4 cm^3^ = 2 points	0 points = 83%
	AVM location	1 point = 79%
	Non-eloquent = 0 points	2 points = 70%
	Eloquent = 1 point	3 points = 48%
	Eloquent = Sensorimotor, language and visual cortex, hypothalamus, internal capsule, brainstem, cerebellar peduncles, and deep cerebellar nuclei	4 points – 39%
	History of hemorrhage	
	No = 0 points	
	Yes = 1 point	

Delayed RIC including neural degeneration can occur after SRS and represented by peri-nidal edema. Depending on the location, patients can be asymptomatic or present with neurologic sequalae that include headache, seizures, and focal weakness (Pollock et al., 2017; Hasegawa et al., 2018; Ilyas et al., 2018). In a recent meta-analysis, the overall rates of radiographic, symptomatic, and permanent RIC were found to be 35.5, 9.2, and 3.8%. Pediatric patients were found to have decreased rates with radiographic RIC in 32.8%, symptomatic RIC in 7.0%, and permanent RIC in 3.2% of patients (Ilyas et al., 2018). Therefore, radiosurgical marginal dose and obliteration rates observed a sigmoid shaped dose-response relationship with a balance between obliteration and adverse radiation effects (Flickinger et al., 1996, 2002). Additional nidal treatment effects of SRS include cyst formation and can be found in ~1–3% of patients at an average of 6.5–7.3 years after treatment (Shuto et al., 2012, 2015). Risk factors that influence cyst formation include higher doses, larger lesions, and lobar locations. Development of cyst are secondary to rupture of delicate telangiectatic nidal vessels after radiation (Chen et al., 2020). Until complete obliteration, the risk of re-bleeding and hemorrhage is unreliable predicted and varies based on lesional size, vascular flow dynamics, and location. Despite this risk, stereotactic radiosurgery remains an essential treatment tool for patients with AVMs.

## AVM biology and development

Brain AVMs are thought to be idiopathic congenital lesions in a developing embryo which present with complications later in life. The pathogenesis and biological development of brain AVMs remains poorly understood, but recent evidence suggests that aberrant angiogenesis may be embroiled in the expansion, development, and rupture of AVMs (Berman et al., 2000; Leblanc et al., 2009; Chen et al., 2014; Rangel-Castilla et al., 2014). Vasculogenesis precedes embryologic cortical folding and studies have found no difference in the cortical folding patterns in normal vs. AVM brains (Shah et al., 2016). Digressive expression of angiogenic factors in central nervous system is a main contributor to vascular malformations including brain AVMs (Mouchtouris et al., 2015). In the surgical specimens derived from brain AVM patients, increased expression of vascular endothelial growth factor (VEGF) in the endothelial cells of AVM nidus vessels has been demonstrated (Hashimoto et al., 2000; Murukesh et al., 2010; Cheng et al., 2019). VEGF expression has potential to up regulated dynamic changes in angiogenesis and expression by nidal tissue can influence AVM formation and resistance to hypoxic factors (Murukesh et al., 2010). Given this, upstream transcription factor signaling networks can influence VEGF and factors such as AKL-1 and can lead to promotion of angiogenesis (Schimmel et al., 2021). Increased soluble endoglin on conjunction with VEGF-A has also been shown to induce dysplastic vessel formation and can influence microglial inflammatory pro-angiogenic endothelial cell dysfunction (Park et al., 2022).

Additionally, arteriovenous specification and vascular stability are regulated by transforming growth factor-β (TGF-β) and its receptors. There are mutations in genes encoding TGF-β signaling molecules which are involved in hereditary hemorrhagic telangiectasia and are also often presented with cranial AVMs. Irregular signaling of TGF-β can cause downstream activation of pro-angiogenic pathways and has been shown to promote cerebrovascular branching and drive angiogenesis (Ferrari et al., 2009; Choi et al., 2012; Cunha et al., 2017; Siqueira et al., 2018; Zhang and Yang, 2020). Additional studies have demonstrated high prevalence of somatic KRAS mutations within blood and tissue derived samples with potentiated mitogen-activating protein kinase pathways (MAPK) and extracellular signal-regulated kinase (ERK) activity. This increased expression of transcription factor mediated changes increases angiogenesis and promotes cellular migration (Cheng and Nussinov, 2018; Nikolaev et al., 2018; Gao et al., 2022). Over expression of such factors as angiopoietin-2 (Ang-2) have been found to regulate angiogenesis and vascular stability (Crist et al., 2019). There is also an elevation in expression of basic fibroblast growth factor b-FGF, interleukin-1β, endoglin, and G protein coupled receptors (Kilic et al., 2000; Lawton et al., 2015).

Dynamic remodeling and nidal growth are known characteristics of AVMs and is influenced by inflammatory specific factors. Genetic inflammatory polymorphisms associated to AVM hemorrhage include interleukin-1β, APOE, and IL-6 (Lawton et al., 2015). Abnormalities in the extracellular matrix of AVMs leads to destabilization of the nidus. Observed changes in metalloproteinases and induced proteolytic degradation can promote structural destabilization and vascular remodeling (Rangel-Castilla et al., 2014).

The altered cellular and structural biology of AVMs deviates from the normal angiogenic principles of cerebral vascular development and regulation of vascular stability. The observed polymorphisms described above promote angiogenesis, influence dynamic remodeling, and destabilize nidal vasculature. Ultimately, this leads to a patient specific lesional characteristics with varying vascular architecture and hemorrhage risk. Radiosurgery for AVMs has proven to be a viable and effect treatment option for AVMs however, changes in the biologic micro-environment are poorly understood.

## Micro-environment changes in AVM after radiosurgery

### General changes in AVM vasculature

The exact mechanism and micro-environmental changes in AVMs in response to radiation has yet to be fully elucidated, though many studies have used *ex-vivo* tissue models, animal models and human histopathology to try and determine the response to radiation (Liu et al., 2012; Simonian et al., 2018; Xu et al., 2018). It is understood that radiation results in cellular damage, particularly to the vasculature endothelium, which demonstrates some of the earliest ultrastructural changes after radiation and are considered the most radiosensitive cells of the vessel wall. Their damage is hypothesized to play a pivotal role in vessel occlusion in AVMs after radiosurgery (Schneider et al., 1997; O'Connor and Mayberg, 2000; Tu et al., 2006; Karunanyaka et al., 2008; Liu et al., 2012; Szeifert et al., 2013). Following separation of the endothelium from underlying vessel wall there is leaking of proteinaceous material into the intimal space (Schneider et al., 1997; Tu et al., 2006, 2009). This is accompanied by proliferation of the subendothelium, smooth muscle cells and spindle cells (Schneider et al., 1997; Sammons et al., 2011; Kashba et al., 2015; Ilyas et al., 2018; Xu et al., 2018; Lee et al., 2019a). These spindle cells have immunohistochemical, ultrastructural and experimental characteristics resembling myofibroblasts, and have contractile capacity through α-smooth-muscle actin production and contribute to vessel occlusion (Sammons et al., 2011; Szeifert et al., 2013; Shoemaker et al., 2020). Smooth muscle cell proliferation occurs with the tunica media of the artery in a circumferential fashion contributing to concentric or eccentric narrowing of the vessel lumen (Schneider et al., 1997; Tu et al., 2006, Figure 1). These smooth muscle cells are found to have Weibel-Palade bodies suggesting a role in protein storage and secretion, such as VEGF, in response to von Willebrand Factor expression post-radiation (Tu et al., 2006). These cell types work synergistically to start the inflammatory and pro-thrombotic process following radiosurgery.

**Figure 1:**
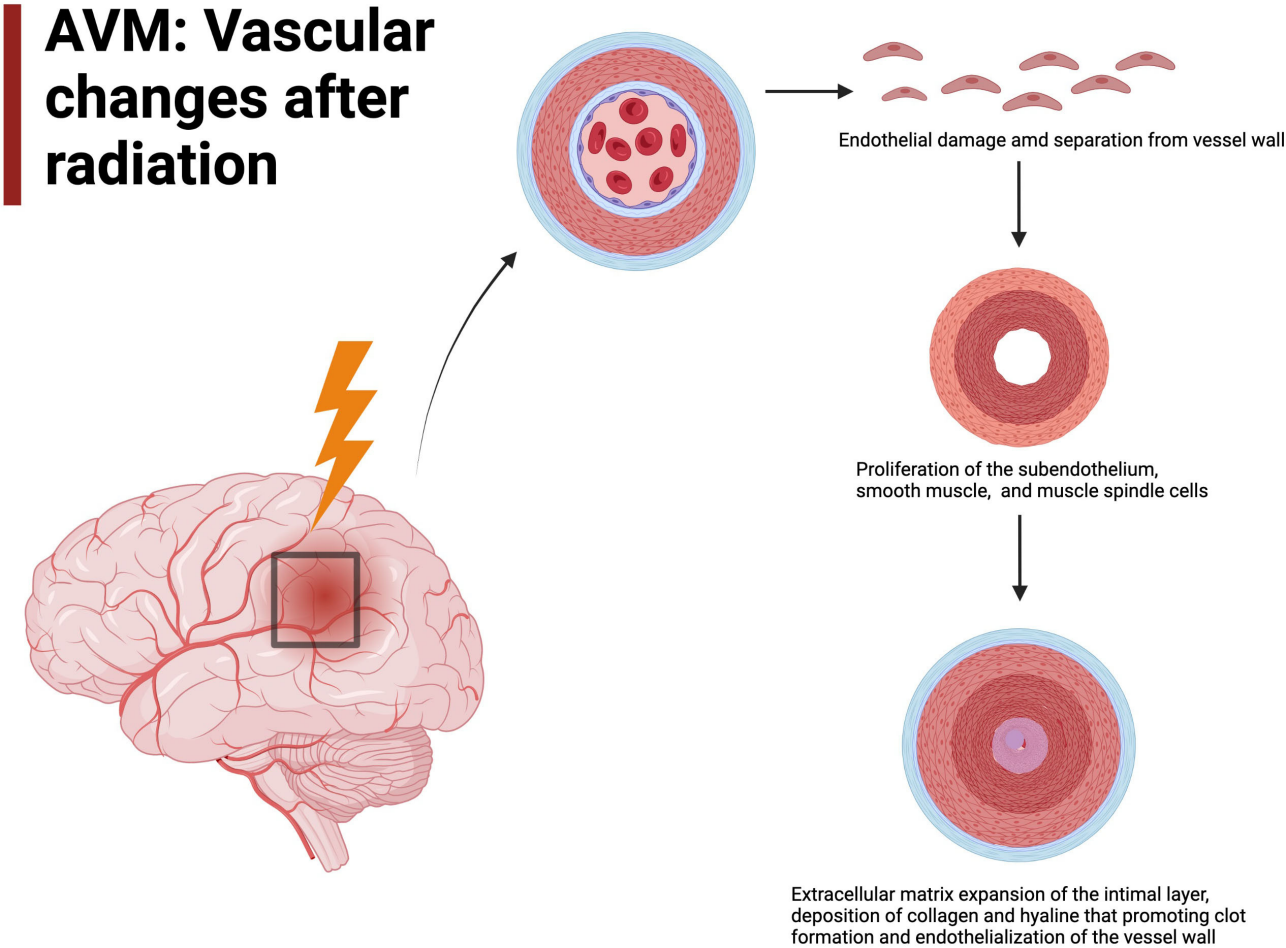
AVM vascular changes after radiation include endothelial damage will lead to endothelialization of the vessel wall.

Following initial cellular degeneration and proliferation, there is extracellular matrix expansion in the intimal layer with deposition of dense fibrillar collagen and hyaline change (Schneider et al., 1997). Fibroblasts and fibrocytes are central to deposition of collagen and clot formation. They produce collagen bundles to replace the myofibroblasts. These changes are slow to occur and several years post-radiosurgery there is evidence of transformation of the initial proteinaceous clots into fibrin thrombi. This is thought to be mediated by growth factors, cytokines, chemokines, and extracellular matrix proteins secreted by fibroblasts and myofibroblasts (Schneider et al., 1997; Tu et al., 2006, 2009). Overtime there is progressive hyalinization of collagen fibers and fibrin thrombi to form scar tissue (Tu et al., 2006; Szeifert et al., 2013). In pathological specimens completely obliterated vessels demonstrate degenerated hyaline scar tissue while incompletely obliterated vessels still have fibrin thrombi indicating this is one of the last steps in the occlusive process in response to radiosurgery (Tu et al., 2006; Szeifert et al., 2013).

Overall, progressive luminal narrowing, medial and intimal thickening, hyalinization and fibrosis occurs leading to intraluminal thrombosis and reduced vessel density due to progressive vascular stenosis (Lo, 1993; Schneider et al., 1997; Tu et al., 2006, 2009; Karunanyaka et al., 2008; Liu et al., 2012; Kashba et al., 2015; Ilyas et al., 2018; Lee et al., 2019a; Xu et al., 2018). Importantly these changes in response to radiation appear to occur in a concentric or eccentric fashion involving all or nearly all of the vessel wall circumference (Schneider et al., 1997). This helps with flow dynamics and vessel wall stress in high flow AVMs. Most data demonstrates the majority of radiation changes occurring no later than 2–3 years after radiosurgery with several studies finding that after 3 years there were no further changes in AVMs in response to radiosurgery. Other studies demonstrate minor changes that continue to occur up to 4–5 years after radiosurgery. Undoubtably, the initial period after radiosurgery is the most critical to obtain AVM occlusion (Chang et al., 1997; Tu et al., 2006, 2013).

### Endothelial structural and molecular changes

In AVMs the endothelium plays a critical role in the pathogenesis of the AVM as well as in its response to radiosurgery. Thus, most research on AVM radiation response has focused on the endothelium and demonstrated a central role in AVM obliteration and vascular remodeling (O'Connor and Mayberg, 2000; Karunanyaka et al., 2008; Xu et al., 2018). There are baseline molecular differences in endothelial cells compared to normal cerebral vasculature, such as increased expression of VEGF, bFGF, TGF-α/β, angiopoietin-2 and NO synthase. These molecular changes suggest a pro-angiogenic process occurring in AVMs compared to normal vasculature. It is unclear if this pro-angiogenic state is part of the primary pathogenesis of AVMs or is secondary to increased shear stress and high flow of the AVM resulting in a pro-angiogenic state (Karunanyaka et al., 2008; Storer et al., 2008; Xu et al., 2018; Lee et al., 2019b). Regardless, evidence demonstrates that AVMs have upregulation of pro-angiogenic molecules which contribute to AVM maintenance. In response to radiation there are several changes to angiogenic molecules that occur. Several studies demonstrate a decrease in angiogenic factors, such as VEGF, TGF-β, and angiopoientin-2, after radiosurgery (Xu et al., 2018; Lee et al., 2019b). These changes were as early as 3 months after radiosurgery with angiopoientin-2 having the greatest reduction immediately after radiosurgery, and well before visible alterations on CT or MRI scans in patients (Xu et al., 2018). Yet, other studies have demonstrated an increase in pro-angiogenic factors after radiation as an initial survival response (Sammons et al., 2011). The exact role of angiogenic factors in AVM occlusion after radiosurgery is not fully elucidated.

**Figure 2:**
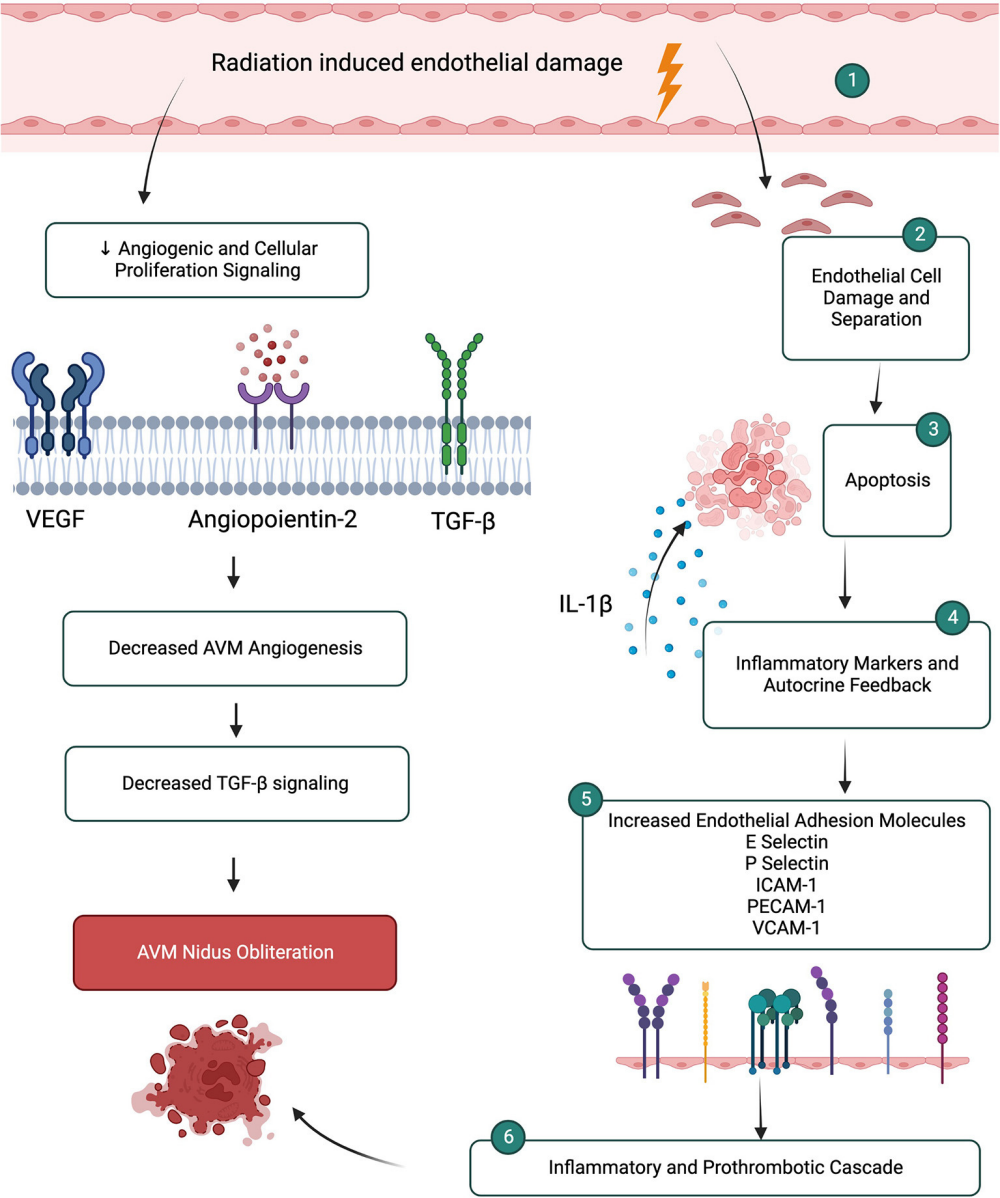
Microenvironment changes after radiation induced endothelial damage leads to inflammatory and prothrombotic cascades promoting AVM nidus obliteration. Simultaneous mechanisms lead to decreased angiogenic and proliferation pathways.

There are several other pro-inflammatory and pro-thrombotic molecular changes that occur in the endothelium in response to radiosurgery. After radiosurgery, endothelial cells are damaged, separate and become disrupted and denuded (Sammons et al., 2011). While undergoing apoptosis endothelial cells release IL-1β which acts as an autocrine positive feedback on apoptotic mechanisms and as a paracrine signal to induce expression of endothelial adhesion molecules and pro-inflammatory cytokines on surrounding endothelial cells (Tu et al., 2013). This importantly starts an inflammatory and pro-thrombotic cascade necessary for AVM obliteration (Karunanyaka et al., 2008). This molecular change starts as early as 4 h after radiation exposure and results in transcriptional upregulation of adhesion molecules E-selectin, P-selectin, ICAM-1, PECAM-1, and VCAM-1 (Karunanyaka et al., 2008; Storer et al., 2008; Sammons et al., 2011; Liu et al., 2012; Tu et al., 2013). While several *in vivo* and *in vitro* studies demonstrate early upregulation of E-selectin within hours of radiosurgery, other studies demonstrate an initial downregulation followed by increased expression (Storer et al., 2008; Liu et al., 2012). The differences in studies may be related to tissue origin of the study, radiation dose, tissue cellular make-up since there is a strong relationship reliance on the subendothelial milieu for E-selectin regulation (Liu et al., 2012). E-selectin and ICAM-1 are upregulated in response to reactive oxygen intermediates generated by radiation that activate NFκB causing increased transcription (Tu et al., 2013). Both, in addition to VCAM-1, are expressed on endothelial cells and facilitate inflammatory cell rolling, adhesion and migration (Tu et al., 2013). P-selectin in stored in Weibel-Palade bodies and mediates leukocyte rolling during inflammation as well as participates in coagulation by binding to tissue factor to accelerate the formation of fibrin during thrombogenesis (Karunanyaka et al., 2008; Tu et al., 2013) (Figure 2).

Pro-thrombogenic molecules are also upregulated in response to radiosurgery in addition to the cell adhesion molecules. Following radiosurgery, the disruption of the endothelial layer results in exposure of the subendothelium which exposes tissue factor, collagen, the basement membrane, von Willebrand Factor, microfibrils and fibronectin which create a procoagulant state (Storer et al., 2007). Importantly, there is exposure of phosphatidylserine, which provides a negatively charged lipid surface for the assembly of coagulation complexes and is necessary for coagulation initiation (Storer et al., 2007, 2010). Phosphatidylserine acts as a co-factor of tissue factor, an important factor in the induction of thrombosis. There is conflicting evidence about the upregulation of tissue factor in response to radiosurgery (Storer et al., 2007, 2010; Liu et al., 2012). Several studies demonstrate an upregulation of tissue factor after radiation that is in a time-dependent manner, yet other studies do not demonstrate a difference in tissue factor expression after radiation (Storer et al., 2007, 2010; Liu et al., 2012). Contrasting evidence also exists for upregulation of von Willebrand Factor and down regulation of thrombomodulin, an anti-coagulation molecule (Storer et al., 2007; Liu et al., 2012). Ultimately, the exact process of intravascular thrombosis after radiosurgery is not well understood but likely involves platelet adhesion followed by thrombus formation due to alteration of pro-thrombotic factors in response to radiosurgery (Karunanyaka et al., 2008).

### Current limitations and future considerations

Ultimately the exact mechanisms of how radiosurgery changes the micro-environment of AVMs and causes obliteration is not understood. Several studies demonstrate that the effects of radiation appear to be a function of vessel size with potentially different responses to radiation dependent on vessel size (Schneider et al., 1997; Storer et al., 2007). Studies also use different experimental AVM models. Human histopathologic studies predominantly look at irradiated AVMs that need to be microsurgically removed secondary to neurologic impairment, and thus are often not AVMs that have been fully responsive to radiosurgery or have been pathologic specimens taken at autopsy many years after radiation exposure. Thus, initial changes after radiation to AVMs in humans is hard to elucidate. Studies use animal models or *ex-vivo* tissue cultures as alternative models for AVM radiation response. Differences in animal physiology, tissue of origin and how tissue culture are prepared can result in different outcomes. As well, it is not difficult to understand how differences in radiation dosing can result in conflicting data in the body of literature (Schneider et al., 1997). Thus, further research needs to be done to fully determine how AVMs respond to radiosurgery at the micro-environmental level.

## Author contributions

TU, KB, AM, and VS were involved in drafting and revision of the original manuscript. TU, KB, AM, and SC were involved in the original conception. TU designed the figure and table. TU, KB, AH, and VS drafted sections of the manuscript. All authors contributed to manuscript revision, read, and approved the submitted version.
